# Supplementary material for: Implications of armed conflict for maternal and child health: A regression analysis of data from 181 countries for 2000–2019
Source: PLoS Med. 2021 Sep 28;18(9):e1003810. doi: 10.1371/journal.pmed.1003810 (PMC8478221; doi:10.1371/journal.pmed.1003810)
Supplement: S1 Table — (DOCX) [file pmed.1003810.s002.docx]

**S1 Table.** Definition of covariates

| **Covariate** | **Type** | **Definition** |
| --- | --- | --- |
| GDP per capita | Continuous | GDP per capita is gross domestic product divided by midyear population. GDP is the sum of gross value added by all resident producers in the economy plus any product taxes and minus any subsidies not included in the value of the products. It is calculated without making deductions for depreciation of fabricated assets or for depletion and degradation of natural resources. Data are in current U.S. dollars. |
| OECD member | Binary | Whether a country is a member of the Organisation for Economic Co-operation and Development. |
| Population density | Continuous | The proportion of people living with a density >1,000 people/km^2^. |
| Urban residence | Continuous | The proportion of people living in urban areas. |
| Age dependency ratio | Continuous | The ratio of dependents--people younger than 15 or older than 64--to the working-age population--those ages 15-64. Data are shown as the proportion of dependents per 100 working-age population. |
| Male education | Continuous | Mean years of educational attainment per capita, males. |
| Temperature | Continuous | Population-weighted mean temperature, degrees Celsius. |
| Rainfall | Continuous | Population-weighted rainfall, mm/yr |
| Earthquakes | Binary | Sudden movement of a block of the Earth’s crust along a geological fault and associated ground shaking. |
| Droughts | Binary | An extended period of unusually low precipitation that produces a shortage of water for people, animals and plants. |
| Electoral democracy index | Continuous | A variable between 0 and 1 formed by multiplying indices measuring freedom of association thick, clean elections, freedom of expression, elected executive, and suffrage. |
| Ethnic Fractionalisation Index | Continuous | The probability that 2 randomly drawn individuals within a country are not from the same ethnic group. |
