# Supplementary material for: Implications of armed conflict for maternal and child health: A regression analysis of data from 181 countries for 2000–2019
Source: PLoS Med. 2021 Sep 28;18(9):e1003810. doi: 10.1371/journal.pmed.1003810 (PMC8478221; doi:10.1371/journal.pmed.1003810)
Supplement: S2 Table — (DOCX) [file pmed.1003810.s003.docx]

**S2 Table.** The association between armed conflict and maternal and child mortality (beta coefficients, 95% confidence intervals) (additional conflict specifications)

|  | **Maternal mortality ratio per 100,000 live births** | | **Under-5 mortality rate per 1,000 live births** | | **Infant mortality rate  per 1,000 live births** | | **Neonatal mortality rate per 1,000 live births** | |
| --- | --- | --- | --- | --- | --- | --- | --- | --- |
| **Battle-related deaths/100,000 population** | 1.25 (-0.68, 3.18) |  | 0.16 (-0.05, 0.37) |  | 0.09 (-0.03, 0.21) |  | -0.00 (-0.05, 0.04) |  |
|  |  |  |  |  |  |  |  |  |
| **Quintile of exposure** |  |  |  |  |  |  |  |  |
| - None |  | 0.00 (0.00, 0.00) |  | 0.00 (0.00, 0.00) |  | 0.00 (0.00, 0.00) |  | 0.00 (0.00, 0.00) |
| - First |  | 7.31 (-11.46, 26.07) |  | -0.01 (-2.74, 2.72) |  | 0.17 (-1.14, 1.49) |  | -0.01 (-0.42, 0.40) |
| - Second |  | 6.03 (-11.34, 23.39) |  | -0.95 (-3.97, 2.07) |  | -0.39 (-1.77, 1.00) |  | -0.32 (-0.86, 0.22) |
| - Third |  | 32.08* (5.71, 58.45) |  | 1.59 (-3.28, 6.47) |  | 1.27 (-0.88, 3.43) |  | 0.10 (-0.60, 0.81) |
| - Fourth |  | 41.07 (-0.62, 82.76) |  | 2.57 (-4.02, 9.15) |  | 1.92 (-1.02, 4.85) |  | 0.14 (-0.89, 1.16) |
| - Fifth |  | 55.83** (16.34, 95.32) |  | 6.42 (-0.79, 13.64) |  | 3.77* (0.21, 7.33) |  | 0.80 (-0.35, 1.94) |
|  |  |  |  |  |  |  |  |  |
| **Covariates** |  |  |  |  |  |  |  |  |
| GDP per capita | 2.86*** (1.60, 4.12) | 2.86*** (1.60, 4.12) | 0.62*** (0.39, 0.86) | 0.63*** (0.39, 0.87) | 0.33*** (0.22, 0.44) | 0.33*** (0.22, 0.45) | 0.11*** (0.07, 0.15) | 0.11*** (0.07, 0.15) |
|  |  |  |  |  |  |  |  |  |
| OECD member | 18.38 (-3.87, 40.62) | 18.29 (-3.52, 40.10) | 3.83 (-2.38, 10.04) | 3.87 (-2.29, 10.04) | 1.53 (-2.35, 5.40) | 1.55 (-2.29, 5.39) | 0.42 (-1.39, 2.23) | 0.43 (-1.37, 2.23) |
|  |  |  |  |  |  |  |  |  |
| Population density | -62.09 (-287.64, 163.47) | -52.65 (-266.69, 161.38) | -39.41 (-102.50, 23.68) | -38.67 (-100.58, 23.24) | -21.40 (-56.26, 13.46) | -20.90 (-55.01, 13.21) | -6.46 (-18.20, 5.27) | -6.28 (-17.75, 5.18) |
|  |  |  |  |  |  |  |  |  |
| Urban residence | -6.71 (-14.04, 0.62) | -6.60 (-13.71, 0.52) | -1.48* (-2.93, -0.02) | -1.45* (-2.88, -0.02) | -0.87* (-1.67, -0.08) | -0.86* (-1.64, -0.08) | -0.35* (-0.65, -0.04) | -0.34* (-0.64, -0.05) |
|  |  |  |  |  |  |  |  |  |
| Age dependency ratio | 0.42 (-1.48, 2.32) | 0.35 (-1.51, 2.20) | 0.15 (-0.16, 0.47) | 0.15 (-0.16, 0.45) | 0.21* (0.04, 0.38) | 0.20* (0.04, 0.37) | 0.13*** (0.06, 0.20) | 0.13*** (0.06, 0.20) |
|  |  |  |  |  |  |  |  |  |
| Male education | -42.27* (-78.03, -6.51) | -44.38* (-79.40, -9.36) | -4.29 (-10.44, 1.86) | -4.45 (-10.71, 1.81) | -1.53 (-4.87, 1.81) | -1.65 (-5.02, 1.72) | 0.25 (-1.12, 1.63) | 0.20 (-1.18, 1.59) |
|  |  |  |  |  |  |  |  |  |
| Temperature | 10.50** (3.98, 17.02) | 11.08*** (4.66, 17.49) | 2.38** (0.95, 3.81) | 2.45*** (1.07, 3.84) | 1.05** (0.31, 1.79) | 1.10** (0.38, 1.81) | 0.24 (-0.04, 0.52) | 0.25 (-0.02, 0.53) |
|  |  |  |  |  |  |  |  |  |
| Rainfall | -4.94 (-14.20, 4.32) | -4.76 (-14.07, 4.56) | 0.49 (-1.24, 2.23) | 0.46 (-1.24, 2.15) | 0.31 (-0.65, 1.26) | 0.30 (-0.64, 1.23) | 0.01 (-0.38, 0.40) | -0.01 (-0.39, 0.37) |
|  |  |  |  |  |  |  |  |  |
| Earthquakes | 5.87 (-2.50, 14.24) | 6.93 (-1.24, 15.09) | 1.58 (-0.43, 3.59) | 1.63 (-0.36, 3.62) | 0.63 (-0.13, 1.39) | 0.66 (-0.08, 1.40) | 0.38* (0.08, 0.68) | 0.40* (0.09, 0.70) |
|  |  |  |  |  |  |  |  |  |
| Droughts | 5.18 (-1.59, 11.96) | 4.56 (-2.28, 11.39) | 1.78** (0.61, 2.95) | 1.77** (0.62, 2.93) | 1.00** (0.37, 1.63) | 1.00** (0.37, 1.62) | 0.39** (0.11, 0.67) | 0.40** (0.12, 0.68) |
|  |  |  |  |  |  |  |  |  |
| Observations | 3,045 | 3,045 | 3,376 | 3,376 | 3,376 | 3,376 | 3,376 | 3,376 |
| Countries | 181 | 181 | 180 | 180 | 180 | 180 | 180 | 180 |

**Note:** * *p* < 0.05, ** *p* < 0.01, *** *p* < 0.001. Robust standard errors were employed. Each column is the output from one panel regression with fixed effects adjusted for the covariates in the table in addition to year dummies (not shown). Coefficients are interpreted as the absolute change in the dependent variable following a change in one unit of the independent variable. GDP per capita is in current US dollars and its unit is scaled up by 1,000. Population density represents the percentage of the population living in a density of >1,000 ppl/sqkm. Urbanisation represents the percentage of the population living in urban areas. The age dependency ratio represents the percentage of the population younger than 15 years and older than 64 years per 100 working-age population. Male education is expressed as years per capita and is age-standardised. Temperature is in degrees Celsius and is the mean population-weighted annual temperature. Rainfall is the mean population-weighted annual rainfall in mm per year, scaled down by 1,000. Earthquake and drought are binary variables representing their absence or presence. All armed conflict variables were lagged by one year.
