# Supplementary material for: Implications of armed conflict for maternal and child health: A regression analysis of data from 181 countries for 2000–2019
Source: PLoS Med. 2021 Sep 28;18(9):e1003810. doi: 10.1371/journal.pmed.1003810 (PMC8478221; doi:10.1371/journal.pmed.1003810)
Supplement: S5 Table — (DOCX) [file pmed.1003810.s006.docx]

**S5 Table.** Sensitivity analysis: sequential addition of covariates, including a complete case analysis (maternal mortality)

|  | Model 1 | Model 2 | Model 3 | Model 4 | Model 5 | Model 6 | Model 7 | Model 8 | Model 9 | Model 10 | Model 11 | Model 12 | Model 13 |
| --- | --- | --- | --- | --- | --- | --- | --- | --- | --- | --- | --- | --- | --- |
| No (<25 battle deaths/year) | 0.00 (0.00, 0.00) | 0.00 (0.00, 0.00) | 0.00 (0.00, 0.00) | 0.00 (0.00, 0.00) | 0.00 (0.00, 0.00) | 0.00 (0.00, 0.00) | 0.00 (0.00, 0.00) | 0.00 (0.00, 0.00) | 0.00 (0.00, 0.00) | 0.00 (0.00, 0.00) | 0.00 (0.00, 0.00) | 0.00 (0.00, 0.00) | 0.00 (0.00, 0.00) |
|  |  |  |  |  |  |  |  |  |  |  |  |  |  |
| Minor conflict (25-99 battle deaths/year) | 19.79 (-0.63, 40.21) | 19.77 (-0.63, 40.18) | 19.02 (-1.23, 39.27) | 19.19 (-1.12, 39.49) | 18.80 (-1.58, 39.17) | 20.19 (-0.51, 40.89) | 20.62 (-0.08, 41.32) | 20.58 (-0.12, 41.29) | 20.59 (-0.09, 41.28) | 20.62 (-0.05, 41.28) | 20.04 (-0.39, 40.48) | 23.71* (2.57, 44.85) | 17.10 (-6.27, 40.48) |
|  |  |  |  |  |  |  |  |  |  |  |  |  |  |
| War (≥1, 000 battle deaths/year) | 36.12 (-1.02, 73.25) | 36.08 (-1.02, 73.17) | 35.01 (-1.34, 71.36) | 33.58 (-2.24, 69.40) | 33.73 (-1.72, 69.18) | 36.56* (1.23, 71.89) | 36.82* (1.62, 72.02) | 36.79* (1.55, 72.02) | 36.89* (1.83, 71.95) | 36.93* (1.86, 72.01) | 34.15 (-0.21, 68.51) | 45.95* (9.50, 82.40) | 36.35 (-1.42, 74.13) |
|  |  |  |  |  |  |  |  |  |  |  |  |  |  |
| **Covariates** |  |  |  |  |  |  |  |  |  |  |  |  |  |
| GDP per capita | 3.78*** (2.40, 5.16) | 3.75*** (2.39, 5.11) | 3.72*** (2.37, 5.07) | 3.41*** (2.10, 4.72) | 3.22*** (1.92, 4.52) | 2.95*** (1.65, 4.26) | 2.90*** (1.62, 4.17) | 2.89*** (1.62, 4.17) | 2.89*** (1.61, 4.16) | 2.89*** (1.61, 4.16) | 3.11*** (1.80, 4.41) | 3.60*** (1.90, 5.30) |  |
|  |  |  |  |  |  |  |  |  |  |  |  |  |  |
| OECD membership |  | 38.67*** (25.40, 51.95) | 34.13*** (17.50, 50.77) | 31.24** (11.37, 51.11) | 23.70* (1.31, 46.09) | 18.80 (-4.87, 42.46) | 19.03 (-3.78, 41.83) | 19.00 (-3.58, 41.58) | 18.39 (-3.63, 40.41) | 18.42 (-3.62, 40.45) | 19.56 (-4.26, 43.38) | 23.47* (2.13, 44.82) | 26.25* (3.43, 49.08) |
|  |  |  |  |  |  |  |  |  |  |  |  |  |  |
| Population density |  |  | -152.82 (-436.89, 131.25) | -95.79 (-351.62, 160.03) | -87.05 (-335.81, 161.71) | -53.43 (-271.18, 164.32) | -56.11 (-276.53, 164.31) | -56.77 (-277.16, 163.62) | -56.48 (-276.79, 163.83) | -56.44 (-276.66, 163.78) | -167.53 (-459.38, 124.31) | -184.63 (-588.06, 218.81) | -16.57 (-215.08, 181.94) |
|  |  |  |  |  |  |  |  |  |  |  |  |  |  |
| Urbanisation |  |  |  | -6.87 (-15.59, 1.84) | -6.88 (-15.04, 1.29) | -6.65 (-13.99, 0.69) | -6.68 (-14.00, 0.64) | -6.69 (-14.00, 0.63) | -6.71 (-14.03, 0.62) | -6.69 (-13.99, 0.62) | -5.28 (-12.14, 1.59) | -2.75 (-11.47, 5.98) | -8.12* (-14.39, -1.85) |
|  |  |  |  |  |  |  |  |  |  |  |  |  |  |
| Age dependency ratio |  |  |  |  | 1.31 (-0.39, 3.01) | 0.48 (-1.39, 2.36) | 0.38 (-1.49, 2.26) | 0.39 (-1.49, 2.27) | 0.41 (-1.48, 2.29) | 0.40 (-1.48, 2.29) | 0.64 (-1.37, 2.65) | -0.84 (-3.19, 1.52) |  |
|  |  |  |  |  |  |  |  |  |  |  |  |  |  |
| Male education |  |  |  |  |  | -42.64* (-78.70, -6.59) | -42.03* (-77.81, -6.24) | -42.16* (-77.99, -6.33) | -42.15* (-78.00, -6.30) | -42.18* (-78.02, -6.34) | -34.15 (-70.12, 1.82) | -45.75* (-90.64, -0.86) | -56.25** (-91.28, -21.23) |
|  |  |  |  |  |  |  |  |  |  |  |  |  |  |
| Temperature |  |  |  |  |  |  | 11.05** (4.49, 17.61) | 10.88** (4.37, 17.38) | 10.83** (4.33, 17.34) | 10.69** (4.22, 17.16) | 9.91** (3.46, 16.36) | 3.96 (-1.54, 9.47) | 15.53*** (7.11, 23.95) |
|  |  |  |  |  |  |  |  |  |  |  |  |  |  |
| Rainfall |  |  |  |  |  |  |  | -4.95 (-14.30, 4.41) | -4.94 (-14.31, 4.42) | -4.35 (-13.78, 5.08) | -8.80 (-20.09, 2.49) | -5.72 (-18.47, 7.03) | -6.06 (-16.02, 3.91) |
|  |  |  |  |  |  |  |  |  |  |  |  |  |  |
| Earthquake |  |  |  |  |  |  |  |  | 5.86 (-2.12, 13.84) | 5.89 (-2.10, 13.88) | 5.74 (-2.33, 13.81) | 2.94 (-5.36, 11.23) | 5.67 (-1.56, 12.90) |
|  |  |  |  |  |  |  |  |  |  |  |  |  |  |
| Drought |  |  |  |  |  |  |  |  |  | 4.73 (-2.10, 11.56) | 4.39 (-2.78, 11.57) | -0.90 (-8.99, 7.19) | 2.89 (-4.27, 10.04) |
|  |  |  |  |  |  |  |  |  |  |  |  |  |  |
| Electoral democracy index |  |  |  |  |  |  |  |  |  |  | -69.48* (-137.50, -1.47) | -66.73 (-135.75, 2.29) |  |
|  |  |  |  |  |  |  |  |  |  |  |  |  |  |
| Ethnic Fractionalisation Index |  |  |  |  |  |  |  |  |  |  |  | 11.60 (-361.87, 385.08) |  |
| Observations | 3,045 | 3,045 | 3,045 | 3,045 | 3,045 | 3,045 | 3,045 | 3,045 | 3,045 | 3,045 | 2,821 | 1,950 | 3,111 |
| Countries | 181 | 181 | 181 | 181 | 181 | 181 | 181 | 181 | 181 | 181 | 168 | 151 | 183 |

**Note:** * *p* < 0.05, ** *p* < 0.01, *** *p* < 0.001. Robust standard errors were employed. Each column is the output from one panel regression with fixed effects adjusted for the covariates in the table in addition to year dummies (not shown). Coefficients are interpreted as the absolute change in the dependent variable following a change in one unit of the independent variable. GDP per capita is in current US dollars and its unit is scaled up by 1,000. Population density represents the percentage of the population living in a density of >1,000 ppl/sqkm. Urbanisation represents the percentage of the population living in urban areas. The age dependency ratio represents the percentage of the population younger than 15 years and older than 64 years per 100 working-age population. Male education is expressed as years per capita and is age-standardised. Temperature is in degrees Celsius and is the mean population-weighted annual temperature. Rainfall is the mean population-weighted annual rainfall in mm per year, scaled down by 1,000. Earthquake and drought are binary variables representing their absence or presence. Electoral democracy index takes a value between 0 and 1, with 1 representing higher democratisation. The Ethnic Fractionalisation Index is the probability that two randomly drawn individuals within a country are not from the same ethnic group. All armed conflict variables were lagged by one year.
