# Supplementary material for: Implications of armed conflict for maternal and child health: A regression analysis of data from 181 countries for 2000–2019
Source: PLoS Med. 2021 Sep 28;18(9):e1003810. doi: 10.1371/journal.pmed.1003810 (PMC8478221; doi:10.1371/journal.pmed.1003810)
Supplement: S6 Table — (DOCX) [file pmed.1003810.s007.docx]

**S6 Table.** Sensitivity analysis: Major Episodes of Political Violence exposure data (maternal mortality)

| **Armed conflict exposure variables** | **Maternal mortality ratio per 100,000 live births** |
| --- | --- |
| No (<25 battle deaths/year) | 0.00 |
| Yes (≥25 battle deaths/year) | 43.18* (1.38, 84.98) |
|  |  |
| **Covariates** |  |
| GDP per capita | 2.86*** (1.61, 4.12) |
|  |  |
| OECD membership | 18.38 (-3.27, 40.02) |
|  |  |
| Population density | -49.94 (-260.67, 160.78) |
|  |  |
| Urbanisation | -6.72 (-13.97, 0.54) |
|  |  |
| Age dependency ratio | 0.45 (-1.43, 2.33) |
|  |  |
| Male education | -40.78* (-75.90, -5.65) |
|  |  |
| Temperature | 10.93*** (4.51, 17.36) |
|  |  |
| Rainfall | -5.65 (-14.82, 3.53) |
|  |  |
| Earthquake | 6.60 (-1.42, 14.61) |
|  |  |
| Drought | 4.77 (-2.01, 11.55) |
| Observations | 3,045 |
| Countries | 181 |

**Note:** * *p* < 0.05, ** *p* < 0.01, *** *p* < 0.001. Robust standard errors were employed. Each column is the output from one panel regression with fixed effects adjusted for the covariates in the table in addition to year dummies (not shown). Coefficients are interpreted as the absolute change in the dependent variable following a change in one unit of the independent variable. GDP per capita is in current US dollars and its unit is scaled up by 1,000. Population density represents the percentage of the population living in a density of >1,000 ppl/sqkm. Urbanisation represents the percentage of the population living in urban areas. The age dependency ratio represents the percentage of the population younger than 15 years and older than 64 years per 100 working-age population. Male education is expressed as years per capita and is age-standardised. Temperature is in degrees Celsius and is the mean population-weighted annual temperature. Rainfall is the mean population-weighted annual rainfall in mm per year, scaled down by 1,000. Earthquake and drought are binary variables representing their absence or presence. All armed conflict variables were lagged by one year.
