# Supplementary material for: Implications of armed conflict for maternal and child health: A regression analysis of data from 181 countries for 2000–2019
Source: PLoS Med. 2021 Sep 28;18(9):e1003810. doi: 10.1371/journal.pmed.1003810 (PMC8478221; doi:10.1371/journal.pmed.1003810)
Supplement: Attachment — Submitted filename: Response to reviewers.docx [file pmed.1003810.s008.docx]

**EDITOR COMMENTS**

**Comment 1**: Please adapt the title to better match journal style. We suggest: "Implications of armed conflict for maternal and child health: A regression analysis of data for 180 countries from 2000-2019".

**Response**: The title has been adapted to match the editor’s suggestion.

**Comment 2**: Please add a new final sentence to the "Methods and findings" subsection of your abstract, which should begin "Study limitations include ..." or similar and should quote 2-3 of the study's main limitations.

**Response**: We have now included the following: “Study limitations include the ecological study design which may mask variation in conflict intensities sub-nationally and the quality of the underlying data.”

**Comment 3**: At line 43, please adapt the sentence to begin "Our analysis indicates that ..." or similar.

**Response**: We have adapted this sentence to match the editor’s suggestion.

**Comment 4**: After the abstract, please add a new and accessible "Author summary" section in non-identical prose. You may find it helpful to consult one or two recent research papers published in PLOS Medicine to get a sense of the preferred style.

**Response**: We have now added an author summary after the abstract.

**Comment 5**: At line 53, please make that "... 5 years".

**Response**: We have edited this sentence accordingly.

**Comment 6**: Early in the methods section of your main text, please state whether the study had a protocol or prespecified analysis plan, and if so attach the relevant document(s) as a supplementary file(s), referred to in the text.

**Response**: We have now stated at the start of our methods the following: “The analysis plan was based on previous conflict and health analytical frameworks developed by the study authors, which included prespecified model structures, covariate selection, and time lags [17]. We used all available maternal and child health outcomes in order to minimise reporting biases.”

**Comment 7**: Please highlight analyses that were not prespecified.

**Response**: All analyses reported in this paper were prespecified, as described in the comment above.

**Comment 8**: At line 96 and any other instances, please make that "data ... were".

**Response**: “Data” has now been pluralised in line 96 and in two other instances in the manuscript.

**Comment 9**: Throughout the text, please format reference call-outs as follows: "... child malnutrition [34,35] ..." (i.e., no spaces in the square brackets).

**Response**: We have now formatted the references accordingly.

**Comment 10**: Throughout the paper, please use the style "up to 2 years", although numbers should be written out at the start of sentences.

**Response**: We have now adopted this style throughout the paper and made numerous changes.

**Comment 11**: Please remove all instances of "[online]" from the reference list.

**Response**: We have now removed all instances of “[online]” from the reference list.

**Comment 12**: Noting reference 13, please ensure that all references have full access details.

**Response**: We have now edited reference 13 to include full access details (volume/page number) and have checked other refences to ensure the same.

**Comment 13**: Please update reference 41, and if not published make the latest version available to us in confidence.

**Response**: We have provided full access details to reference 41, which is now published.

**Comment 14**: Please add a completed checklist for the most appropriate reporting guideline, which may be RECORD, labelled "S1_RECORD_Checklist" or similar and referred to as such in the Methods section.

**Response**: This checklist has been added as a supplementary file.

**Comment 15**: In the checklist, individual items should be referred to by section (e.g., "Methods") and paragraph numbers, not line or page numbers as the latter generally change in the event of publication.

**Response**: The checklist has been completed in line with the editor’s suggested approach.

**REVIEWER 1**

**Comment 1**: Did the authors consider a measure of accessibility to healthcare or healthcare systems as a potential confounder?

**Response**: Thank you for this suggestion. We did consider this and data on accessibility to healthcare and healthcare quality/performance were available to us for possible inclusion in this study. However, on reviewing prior conceptual and theoretical frameworks we considered healthcare variables as mediators rather than confounders. We consider a confounder as a pre-exposure covariate that unidirectionally causes an exposure (in this case, conflict) and also causes an outcome (in this case, mortality). While it is plausible that reduced accessibility to healthcare can cause conflict (for example, through increased social unrest), it is arguably a stronger relationship the other way round (i.e. conflict causes reduced accessibility to healthcare). Therefore, we consider healthcare accessibility on the causal pathway between armed conflict and mortality, and as mediators do not require adjustment in models testing this pathway.

**Comment 2**: Did the authors consider completing a multiple imputation methodology for missing data for comparison, to further assess the robustness of their findings?

**Response**: In general, we did not find missingness to be a major problem for the analyses and do not believe that patterns of missing data bias the results substantially. Eight out of 12 covariates used in this study were 100% complete. As mentioned in the paper, missingness in the 4 other covariates was: GDP per capita (2.7%), age dependency ratio (3.9%), Electoral Democracy Index (11.8%), and Ethnic Fractionalisation Index (46.0%). The latter two were omitted from the models due to high levels of missing data, but reintroduced in sensitivity analyses. All exposure and outcome variables were 100% complete except the secondary outcome skilled birth attendant which had 55.9% missing. Thus two variables had missingness <5% and three had missingness >10%.

For variables with missingness <5%, we originally considered mean imputation techniques, which are suitable if less than 10% of the data are missing [1], and found the effect sizes to be similar to complete case analyses. Therefore, to promote clarity of the messages in the paper and because multiple imputation would have offered little benefit, we decided not to present analyses with multiple imputation. Additionally, multiple imputation assumes that the data are missing at random, which was not the case in our dataset. For example, GDP was missing for entire countries (e.g. Taiwan, North Korea) rather than being missing in a random pattern across country-year observations. Therefore, we felt the presentation of multiple imputation results were even less appropriate. However, should the reviewers or editors wish, we can present these data in a supplementary material.

For the variables with high missingness, we opted to be clear in the methods and either omit and test with sensitivity analyses or conduct sub-group analyses (skilled birth attendance). Given the high missingness, multiple imputation would have been inappropriate here.

[1] Lodder, P. To impute or not to impute: that’s the question. *Advising on Research Methods: Selected topics.* 2013;1-7.

**REVIEWER 2**

**Comment 1**: Line 137: The authors made a decision to not include countries that have many minor conflicts summing to more than 1000 battle-related deaths per year with the countries that have experienced war. It would be helpful it the authors could provide more information as to what they mean by multiple minor conflicts and to provide a rationale for this distinction. If there are many minor conflicts in a country that amount to the same number of deaths, why would this not have a similar impact as a war on the health system or macro-level environment, thus leading to the same outcomes as hypothesized resulting from exposure to war? Did the authors perform any sensitivity analysis to interrogate this decision?

**Response**: We thank the reviewer for this excellent point. The main rationale for our decision was to prevent large countries with many small conflicts (e.g. India) being classified as war-affected. We investigated the data further and found that if we were to go with the reviewer’s suggested classification, 68/522 observations would be reclassified from minor conflict to war. We re-ran our regression models with this classification and found a very small increase in effect sizes but not enough to affect the statistical significance of any results. We will therefore continue with the original classification. We have now added the following sentences to the methods section of the revised manuscript to explain this rationale:

“The rationale behind this decision was to prevent bias where large countries with many minor conflicts, such as India, were incorrectly classified as war-affected. This decision meant that 68 country-year observations were categorised as experiencing minor conflicts despite having more than 1,000 battle-related deaths, and we found no difference in models that categorised these 68 country-year observations as minor conflict or war, so we opted for the former.”

We would also like to highlight to the reviewer that this classification only affects one of our four armed conflict specifications (the categorical model), and so broadly speaking has very minimal impacts on our results in general.

**Comment 2**: In the methods, some of the co-variates are not fully defined. For example, the authors do not define how skilled birth attendance is measured. Authors should make sure that all exposures and covariates are clearly defined.

**Response**: We have now defined skilled birth attendance in the main text and have included an additional table appendix with definitions of all covariates (S1 Table). We have also shortened the paragraph on covariates, some of which includes definitions of selected covariates, as they are now all found in S1 Table.

**Comment 3**: As stated above, the discussion section presents a rather simplistic interpretation of the results and focuses primarily on reduced government spending on healthcare and damage to healthcare infrastructure. However, there are several other factors that could contribute to the increases observed and be equally important. For example, conflict likely limits a woman's ability to travel to a facility for ANC and delivery; though of note, it is interesting that the authors do not find a significant decrease in skilled birth attendance associated with conflict. To my earlier point, a clearer definition of what skilled birth attendance means in this study would be helpful to develop a more nuanced picture of the results. Are the authors able to further unpack skilled birth attendance by looking at institutional births or cadre of "skilled" attendant? The authors do state that this finding could be the result of a type II error, though it is noted that the confidence intervals around the point estimates are not particularly wide. Providing some deeper discussion here could add depth to the interpretation of these results.

**Response**: We have now defined skilled birth attendant in the main text and expanded our discussion to better explain our findings. We have drawn further on the literature, including the BRANCH Consortium publications recommended by Reviewer 3 (see Comment 8) to provide a more nuanced interpretation. Unfortunately the data did not allow for a disaggregation of the skilled birth attendant variable into cadres, nor were there variables available on institutional delivery. We have added the following to the methods to highlight the degree of missing data in the skilled birth attendant variables:

“There were missing data in the skilled birth attendant outcome measure (55.9%), but it was included in the analysis to minimise reporting bias.”

We have also included an additional paragraph that provides a deeper discussion around skilled birth attendants:

“An established literature links armed conflict to lower workforce retention and increased job stress and insecurity [36,37], but this study did not find significant associations between conflict and changes in skilled birth attendance. Reductions in workforce retention may be partly offset by increased humanitarian support, particularly in the short term. For example in Syria, humanitarian agencies were able to successfully advocate funding for prioritisation of reproductive and maternal healthcare [38]. Research from Afghanistan showed that coverage of skilled birth attendants and institutional facility deliveries continued to increased linearly between 2003 (~20%) and 2015 (~50%), explained by early investment in maternal health programmes [33]. A similar pattern was recently documented in Colombia [39]. Variation in sociocultural factors between countries may also partly explain our findings. For example, research conducted among Syrian refugees in Lebanon found that expectant mothers preferred to return to war-torn Syria for planned Caesarean sections to prevent against uncertainties in reaching a hospital during natural labour (e.g. blocked roads, night curfews, etc) [40]. It is also plausible that these results are driven by a selection bias given over half of the global data were missing for skilled birth attendance and missing data were more frequent in conflict-affected countries where data availability and quality are more limited.”

**Comment 4**: Further, the authors do not reflect on some of their more interesting findings in the discussion. For example, it is quite interesting that under-5 mortality increases only between three and eight years since the onset of war. Why might this be the case and why might the effects not be immediately observed?

**Response**: We have now added the following paragraph that discusses our findings from lagged models.

“This study highlights how the negative health impacts of conflict may appear and persist several years after the onset of war, particularly with respect to under-5 mortality. Delayed impacts of armed conflict may reflect an amalgamation of several upstream and healthcare factors conducive to good health, such as the withdrawal of humanitarian funding, the slow breakdown of vital health and social infrastructure, and the accumulation of risk factors in pre-birth and in early life, including delays to immunisation, diarrhoea, and the absence of antibiotics [48].”

**Comment 5**: Similarly, I think one of the more important findings of this paper is the significant lagged effects observed with regard to several of the outcome variables. While the authors reference this finding in their conclusions; they give little interpretation of these results in the discussion section.

**Response**: We have now included several sentences to discuss our findings on lagged effects, as per the previous comment (see Discussion, sixth paragraph).

**Comment 6**: Given that other studies have recently looked at maternal mortality (several of which are cited), and other related indicators, such as institutional delivery, using the same database to assess conflict exposure, it would be helpful if the authors could contextualize the results of this study in the existing literature.

**Response**: We have now reflected on a wider literature throughout the discussion section, supported by the BRANCH Consortium publications highlighted by Reviewer 3. Please see our response to Comment 3 above which includes an updated comparison to the literature.

**Comment 7**: The authors mention in the limitations that "DHS does not provide a representative picture of conflict exposure and so “can over- or underestimate the effects of conflict depending on the sampling approach [39]. Additionally, DHS analyses are limited in their ability to assess trends and impact of shocks such as conflict [40]- which is a key strength of this study." These statements are not clear. In particular, DHS does not measure conflict and is not conducted during times of conflict, thus it is not clear how the DHS would under or over-estimate the effects of conflict. Further, it is not clear what the authors are referring to their improved ability over these other studies to "assess trends and shocks such as conflict," given that the data used to measure conflict is this analysis is the same as that used in the analyses that use DHS. More clarity here is needed. The papers referenced by the authors. Further, these statements are not appropriately referenced.

**Response**: Thank you for highlighting this lack of clarity. We have now rephrased this part of the discussion and it now reads:

“However, the UN Maternal Mortality Estimation Inter-agency Group cautions against using DHS data for monitoring trends as its methodology (asking respondents about survivorship of sisters) generates estimates pertaining to any time within the past few years before the survey [50]. A key strength of our study is the use of data from the World Bank which, unlike the DHS, enables an assessment of trends and the impacts of shocks such as armed conflict.”

**Comment 8**: Reference 39 is not directly relevant to the DHS while reference 40 appears to have been made in error.

**Response**: Reference 40 (now 50) was the correct reference, as the World Bank data page provides a cautionary note about the use of DHS data (one must click on the “details” box, located at the top right of the graph, which produces a pop up box with the note, but doing so does not change the URL).

**Comment 9**: Given that several other papers have examined conflict exposure using smaller scale geographies, and this paper takes a country-level ecological approach, it would be helpful to see a more nuanced discussion of why this approach is particularly meaningful, what new can be learned from it beyond what has already been published, etc. beyond the little that is mentioned in the limitations section.

**Response**: We have now added the following the first paragraph of the discussion:

“We provide powerful and new global estimates of conflict impacts beyond limited geographies of previous work, taking an approach that adjusts for country-level factors and explores time trends while accounting for pre-conflict and post-conflict trends.”

**Comment 10**: Page 21 (no line numbers): what do the authors mean by "data manipulation?" This is not clear.

**Response**: We thank the reviewer for raising this point about lack of clarity. All data provided by organisations such as the World Bank, World Health Organisation, Institute for Health Metrics and Evaluation, etc, are cleaned and edited prior to publication to account for data quality issues such as missing and anomalous data. We have now rephrased from “…biases introduced by data manipulation or missing data…” to “biases introduced by estimation and data processing of data sources before publication (e.g. WHO or IHME)”

**Comment 11**: In conclusion, the authors state "This study shines light on the major challenges that countries in conflict face in meeting SDG targets for maternal and child health, especially in settings where conflict is of high intensity and long lasting." I do not see where the authors examine interactions between duration and intensity of conflict. It would seem that this would be a particularly salient dimension to explore—especially given that the effect of prolonged conflict on systems-level deterioration may compound over time as opposed to conflicts that are more brief in duration.

**Response**: This is an important point. We appreciate we did not assess conflict duration in this paper and so have removed “long lasting” from the conclusion. We agree with the reviewer that this is an important dimension to explore, however we believe this would have been a complex analysis to introduce into the paper and would have crowded other messages. To explore the effects of conflict duration we believe a robust and comprehensive analysis specifically on this topic would be better rather than short supplementary analysis in this paper. For example, duration could be measured through lagged effects over different years, or a cumulative variable denoting total years of conflict. It may be appropriate to adapt the analytical framework and analytical strategy to consider more robustly the temporal nature of any associations. To acknowledge this better, we have noted it as a limitation in our discussion: “The role of conflict duration was not incorporated into the analytical approach, and this should be considered as an additional dimension to assessing conflict impacts in future work.”

**REVIEWER 3**

**Comment 1**: Introduction: I suggest to add a paragraph that introduce some of the other variables associated with the outcomes of interest that are taken into account as potential confounders in the analysis and how some of this are highly correlated with conflict.

**Response**: Thank you for this suggestion. We have now added the following paragraph to the introduction:

“For example, one major critique of the conflict literature is its approach to confounding variables. A review of 46 quantitative studies by Dixon found over 200 independent variables had been assessed as possible causes of war, with a high degree of consensus in no more than four (demographics, economy, history of insecurity, and democratisation) [14]. As many independent variables may be collinear with armed conflict, it highlights the importance of grounding conflict models in conceptual frameworks and measuring collinearity between variables.”

**Comment 2**: Methods: I suggest expanding the explanation of the population mean temperature and rainfall, it is not very clear.

**Response**: We have now included S1 Table which has the definitions of all covariates.

**Comment 3**: Page 9, statistical analysis: "Four data points (Rwanda 1994, Bosnia and Herzegovina 1995, Congo 1997, and Eritrea 1999) were considered outliers due to having high leverage 184 and influence, and were omitted from the model.." please expand or clarify exactly to what variable are you referring.

**Response**: The dataset is organised in such a way that each observation (data point) is a country-year. Following our review of outliers we dropped four observations, or all variables for four country-years. We have now used the term “observation” instead of “data point” to help better clarify this sentence.

**Comment 4**: Results: Table 2: improve the title to include that it is an adjusted model.

**Response**: We have included the term “adjusted beta coefficients” in the title of Tables 2 and 3.

**Comment 5**: It seems that there is a mistake in the number of the figure captions.

**Response**: We have checked the figure captions again but could not find the mistake described by the reviewer.

**Comment 6**: Is there a way to account for possible correlation or collinearity between conflict and confounding variables?

**Response**: We ran post-regression diagnostics which included the variance inflation factor (VIF) to identify collinear variables. We have included the following sentence to highlight collinearity: “We found no collinearity in our models (VIF<2).”

**Comment 7**: I suggest including a brief description of the findings related to the confounding variables in the models.

**Response**: Our primary and secondary analyses include 28 separate models and therefore we felt it was not possible to report findings related to confounding variables without considerable lengthening of the manuscript. There could also be challenges interpreting the effect sizes of coefficients as these are to adjust for potential confounding between conflict and health outcomes rather than being interpretable on their own [1].

[1] Westreich D., & Greenland S. The Table 2 Fallacy: Presenting and Interpreting Confounder and Modifier Coefficients. *Am J Epi.* 2013;177(4):292-298

**Comment 8**: Discussion: The Branch consortium has a series of recent publications that could help to expand the comparisons with other studies. <https://www.biomedcentral.com/collections/branchconsortium>

**Response**: Thank you for highlighting this series, we have now cited many of these publications throughout the discussion (please see our above response to Reviewer 2, Comment 3).
