# Supplementary material for: Implications of armed conflict for maternal and child health: A regression analysis of data from 181 countries for 2000–2019
Source: PLoS Med. 2021 Sep 28;18(9):e1003810. doi: 10.1371/journal.pmed.1003810 (PMC8478221; doi:10.1371/journal.pmed.1003810)
Supplement: Attachment — Submitted filename: Response to reviewers R2.docx [file pmed.1003810.s009.docx]

**EDITORS’ COMMENTS**

**Comment 1**: Please address the issues of selection bias and missing data for each of the main outcomes (with summary data on each provided for review)

**Response**: Please find below summary data for each primary and secondary outcome:

| Outcome | Years covered | Observations (n) | Countries (n) | Missing (%) | Source |
| --- | --- | --- | --- | --- | --- |
| Primary outcomes | | | | | |
| Maternal mortality ratio | 2000-2017 | 3,294 | 183 | 0.0 | World Bank |
| Under five mortality rate | 2000-2019 | 3,700 | 185 | 0.0 | World Bank |
| Infant mortality rate | 2000-2019 | 3,700 | 185 | 0.0 | World Bank |
| Neonatal mortality rate | 2000-2019 | 3,700 | 185 | 0.0 | World Bank |
| Secondary outcomes | | | | | |
| Deliveries by a skilled birth attendant | 2000-2018 | 1,692 | 184 | 52.6 | World Bank |
| DPT vaccination coverage | 2000-2019 | 3,681 | 185 | 0.5 | World Bank |
| Measles vaccination coverage | 2000-2019 | 3,681 | 185 | 0.5 | World Bank |

We now include the following under the methods section:

“Data were fully complete for all primary outcomes while missing data were present in 52.6% of observations for skilled birth attendance and 0.5% of observations for both DPT and measles vaccination coverage. For deliveries by a skilled birth attendant, data were not missing at random. The number of observations varied from 10 to 135 countries per year, with fewer observations after 2015. Observations during armed conflict constituted 20.6% (n=443/2148) of missing data but 13.4% (n=227/1692) of non-missing data, suggesting selection bias should be considered when interpreting these findings.”

For DPT and measles vaccination coverage, data are not missing at random. The 19 missing observations are from Montenegro (2000-2005), Timor-Leste (2000-2001), and South Sudan (2000-2010). All three countries were not in armed conflict during these years.

**Comment 2**: We ask you to add discussion to put your findings in context of other related results from the BRANCH and PRIO consortia.

**Response**: This comment was also raised by Reviewer 3 in the previous round, and in response we cited six additional references from the BRANCH Consortium (Mirzazada *et al.*, Tyndall *et al*., Ataullahjan *et al.*, Sami *et al.*, Akik *et al.*, and Ramos-Jaraba *et al.*). Apologies that this was not made clear in our previous response to Reviewer 3. The manuscript also contains four further references from authors of the BRANCH Consortium, including Bendavid *et al.*, Wagner *et al*. (2018 and 2019), and Wise *et al*. We checked the BRANCH Consortium publication list at <https://www.branchconsortium.com/search> and we believe that we have cited the most relevant articles in our discussion.

We also searched publications of the PRIO Consortium at <https://www.prio.org/Publications/Publications/> to find additional references above the three already included from this group (Urdal & Che, Ostby *et al.*, and Plumper & Neumayer). We have now cited an additional five papers in the introduction and discussion sections (Kotsadam & Ostby, Kotsadam *et al.* and Chi *et al*. [two 2015 articles, one 2018 article]).

We have now made the following additions to the discussion section:

“Further empirical evidence from Nigeria has found that living in close proximity to development aid during conflict is associated with lower infant mortality [15].”

“Furthermore research from conflict-affected Burundi showed that integrating traditional birth attendants into the primary health care system as “birth companions” encouraged expectant mothers to seek healthcare rather than give birth at home [46].”

**Comment 3**: We also suggest devoting some discussion to the findings on deliveries with skilled birth attendants and neonatal mortality, where the observed impacts may be less than would be expected.

**Response**: We have now included a paragraph in the discussion section interpreting our findings with respect to neonatal mortality and skilled birth attendance:

“The absence of a significant relationship between conflict and neonatal mortality indicates that mechanisms underlying maternal and child health impacts may be complex and vary between outcome measures. One interpretation is that birth is a key event for which mothers are most likely to seek care, even in fragile and insecure environments, which coupled with the skilled birth attendants could mitigate against the risk of neonatal mortality. In contrast, conflict may dissuade mothers from seeking healthcare during pregnancy and infancy, explaining increases in maternal and infant mortality and reduced vaccination uptake. This is supported by research conducted among Syrian refugees in Lebanon, which found that expectant mothers preferred to return to war-torn Syria for planned Caesarean sections to prevent against uncertainties in reaching a hospital during natural labour (e.g. blocked roads, night curfews, etc) [45]. Furthermore research from conflict-affected Burundi showed that integrating traditional birth attendants into the primary health care system as “birth companions” encouraged expectant mothers to seek healthcare rather than give birth at home [46]. It is also plausible that our results for skilled birth attendants are affected by selection bias given over half of the global data were missing for skilled birth attendance and missing data were more frequent in conflict-affected countries where data availability and quality is limited.”

**Comment 4**: Please adapt the data statement (submission form) to provide the links where study data can be obtained.

**Response**: We will provide links for the data statement on the submission system.

**Comment 5**: Thank you for describing the origin of your analysis plan. Are you able to attach a document or documents as a supplementary file, referred to in the methods section?

**Response**: The analysis plan originates from the PhD thesis of the first author which is currently under embargo, but we have cited another publication from this thesis in the main text of the methods section which sets out the common analytical frameworks, prespecified model structures, covariate selection, and time lags used (see reference 19, Jawad *et al.*)

**Comment 6**: Please use the journal name abbreviation "PLoS ONE" in the reference list; and use other abbreviations, e.g., "Confl Health", consistently.

**Response**: We have checked the references and abbreviated journal names consistently.

**REVIEWER 2**

**Comment 1**: The authors mention that over half of the countries are missing data on SBAs and that more conflict-affected countries are missing data on this variable than non-conflict affected countries. This seems like a substantial limitation in the paper and it is difficult for me to understand how this offers a robust estimate of the association given the extent of missing data, but the authors only make a passing comment about this issue. Are any of the other variables of interest missing data? It would be helpful for the authors to provide this additional information for other important variables in the paper that may also be subject to selection bias in this same way due to missing data.

**Response**: Please see our response to Comment 1 of the Editors’ Comments above. A description of missing data is now included more comprehensively across all outcome variables.

**Comment 2**: The authors discuss the limitation with the DHS data as it being unable to assess trends. "A key strength of our study is therefore the use of data from the World Bank, which unlike the DHS, enables an assessment of trends and the impacts of shocks such as armed conflict." However, the limitation that they describe really only seems to be relevant to estimates of maternal mortality given the DHS use of the sisterhood method, and it is hard to understand how this would apply to other other outcomes of interest investigated by the authors. The authors should be more precise in discussing these limitations.

**Response**: We agree with the reviewer that the sister survivorship method of the DHS is only relevant to maternal mortality, and we have made this clear by including “on maternal mortality” to the sentence cited by the reviewer.
