# Supplementary material for: Implications of armed conflict for maternal and child health: A regression analysis of data from 181 countries for 2000–2019
Source: PLoS Med. 2021 Sep 28;18(9):e1003810. doi: 10.1371/journal.pmed.1003810 (PMC8478221; doi:10.1371/journal.pmed.1003810)
Supplement: Attachment — Submitted filename: Response to reviewers R3.docx [file pmed.1003810.s010.docx]

**EDITORS’ COMMENTS**

**Comment 1**: At line 31, prior to the presentation of the findings, we suggest adding a sentence to summarize the number of observations, number of minor conflicts and number of wars, for example.

**Reply**: We have now included the following at line 31: “Out of 3,718 country-year observations, 522 (14.0%) were minor conflicts and 148 (4.0%) were wars.

**Comment 2**: Parenthetically, why are 192 countries specified in table 1 when you highlight 180 countries in the title etc?

**Reply**: Thank you for highlighting this inconsistency, which is due to Table 1 incorrectly including 6 countries without any mortality data but with conflict and covariate data. For clarification, maternal mortality data were available for 183 countries between 2000-2017 while under-five/infant/neonatal (“child”) mortality data were available for 185 countries between 2000-2019. Andorra, Dominica, and the Marshall Islands had data for child mortality but not for maternal mortality. Conversely, Puerto Rico had data for maternal mortality but not for child mortality. Therefore, there are 186 unique countries with either maternal or child outcome data, not 192, and the total number of observations is: (185 countries x 20 years) plus (18 maternal mortality years from Puerto Rico) = 3,718. This has been reflected in an updated Table 1 and does not affect the remaining results or tables.

The number of countries drops to 181 for maternal mortality and 180 for child mortality once adjusted for confounders (due to missing data in GDP and the age dependency ratio), and we use the number 180 in the title etc to reflect the number of countries included in the child mortality regression models, which are our main results.

**Comment 3**: At line 36 and elsewhere in the paper, please avoid the word "effects" given the observational study design, substituting "impacts" or similar.

**Reply**: We have replaced “effects” and other similar causal language throughout the manuscript.

**Comment 4**: At line 36 we suggest amending the wording to: "Long term impacts of war were demonstrated by associated increases in maternal mortality observed for up to 7 years; in under 5 mortality ..." or similar.

**Reply**: The wording has been amended accordingly.

**Comment 5**: At line 46, "... relating to"?

**Reply**: Thank you for spotting this typo, we have changed it to “relating to”.

**Comment 6**: Please spell out "DPT" at first use in abstract, author summary and main text.

**Reply**: Thank you, we have now written this out in full in the abstract, author summary, and main text.
